# Expression profiling *in vivo *demonstrates rapid changes in lung microRNA levels following lipopolysaccharide-induced inflammation but not in the anti-inflammatory action of glucocorticoids

**DOI:** 10.1186/1471-2164-8-240

**Published:** 2007-07-17

**Authors:** Sterghios A Moschos, Andrew E Williams, Mark M Perry, Mark A Birrell, Maria G Belvisi, Mark A Lindsay

**Affiliations:** 1 Biopharmaceutics Research Group, Airway Diseases Unit, National Heart and Lung Institute, Imperial College, London SW3 6LY, UK; 2 Respiratory Pharmacology, Airway Diseases Unit, National Heart and Lung Institute, Imperial College, London SW3 6LY, UK

## Abstract

**Background:**

At present, nothing is known of the role of miRNAs in the immune response *in vivo *despite the fact that inflammation is thought to underlie multiple acute and chronic diseases. In these circumstances, patients are commonly treated with corticosteroids such as dexamethasone.

**Results:**

To address this question, we have examined the differential expression of 104 miRNAs using real-time PCR during the innate immune response in mouse lung following exposure to aerosilised lipopolysaccharide (LPS). Following challenge, we observed rapid and transient increase in both the mean (4.3-fold) and individual levels of miRNA expression (46 miRNAs) which peaked at 3 hrs. Crucially, this increase was correlated with a reduction in the expression of tumour necrosis factor (TNF)-α, keratinocyte-derived chemokine (KC) and macrophage inflammatory protein (MIP)-2, suggesting a potential role for miRNAs in the regulation of inflammatory cytokine production. Examination of the individual miRNA expression profiles showed time dependent increases in miR-21, -25, -27b, -100, 140, -142-3p, -181c, 187, -194, -214, -223 and -224. Corticosteroid studies showed that pre-treatment with dexamethasone at concentrations that inhibited TNF-α production, had no effect either alone or upon the LPS-induced miRNA expression profile.

**Conclusion:**

We have shown that the LPS-induced innate immune response is associated with widespread, rapid and transient increases in miRNA expression in the mouse lung and we speculate that these changes might be involved in the regulation of the inflammatory response. In contrast, the lack of effect of dexamethasone in either control or challenged animals implies that the actions of glucocorticoids *per se *are not mediated through changes in miRNAs expression and that LPS-induced increases in miRNA expression are not mediated via classical inflammatory transcription factors.

## Background

RNA interference (RNAi), mediated by 19- to 23- nucleotide double stranded RNA, has been identified as an evolutionarily conserved mechanism for the post-transcriptional regulation of mammalian gene expression [1]. Initial studies implicated RNAi in the cellular response to viral infection following the demonstration that virally-derived double stranded RNA was cleaved by the action of the RNase-III-type enzyme Dicer, to produce the characteristic RNA duplexes. These were called short interference RNA (siRNA) in order to differentiate them from longer double stranded RNA (> 30 nucleotide duplexes) that induced an interferon-mediated anti-viral response. The biological action of siRNAs is mediated via the RNA-induced silencing complex (RISC) that utilises the guide strand as a template to cleave the complementary mRNA sequence and attenuate subsequent protein production [2].

Significantly, recent studies have also identified the existence of endogenous genes that are converted to siRNA-like molecules, entitled microRNAs (miRNA) [3,4]. These genes are transcribed by RNA polymerase II [5,6], processed by the RNase III enzyme Drosha in combination with DGCR8 to produce a hairpin RNA of ~65-nucleotides [7] before being exported into the cytoplasm by exportin 5 [8]. Like siRNAs, these pre-miRNAs are then cleaved by Dicer to produce 19- to 23-nucleotide RNA duplexes, which are incorporated into a RISC-like complex. However, although miRNAs are also able to induce mRNA cleavage [9,10], they are believed to predominately block either mRNA translation or promote degradation following imperfect complementary binding of the guide sequence to miRNA-recognition elements (MRE) within the 3' untranslated region (UTR) of target genes [11-15]. Significantly, within the guide strand the specificity is thought to be primarily mediated by the 'seed' region localised at residues 2–8 at the 5' end [16] although recent studies have shown that their biological activity is influenced by a number of additional factors including the number of MRE binding sites [17], the distance between MRE binding sites [18] and the higher order structure of the target mRNA [19].

Although nearly 500 miRNAs have been identified in rats, mice and humans their physiological role is still uncertain [20,21]. As a result of the redundancy within the 'seed' region, predictive algorithms have suggested that up to a third of all genes may contain putative single or multiple MREs and might therefore be regulated by changes in miRNA expression [22]. Early functional studies have demonstrated a role for miRNAs in apoptosis/proliferation [23,24], development [25-27], insulin secretion [28] and cholesterol metabolism [29,30] whilst differential expression studies have shown a possible role in multiple cancers [31,32].

The innate immune response mediated by epithelial cells and immune cells such as macrophages, neutrophils and dendritic cells, is the first line of defence against infection. This response involves the recognition of conserved molecular patterns upon the invading micro-organism, entitled pathogen-associated molecular patterns (PAMPs), by members of the Toll-like receptor (TLR) family. At present, eleven members of the TLR family have been identified, including TLR-4 which recognises lipopolysaccharide (LPS) derived from gram negative bacteria. Importantly, exposing animals to LPS has been shown to stimulate a host of responses including the release of pro-inflammatory chemokines and cytokines [33,34] which is mediated through activation of intracellular signalling pathways involving TRAF-6/IRAK-1 and recruitment of pro-inflammatory transcription factors such as nuclear factor (NF)-κB and activator protein (AP)-1 [35,36].

Glucocorticoids are commonly used to treat the inflammation associated with many acute and chronic diseases including asthma, rheumatoid arthritis, auto-immunity, psoriasis and multiple sclerosis. Under resting conditions, the glucocorticoid receptor (GR) is localised within the cytoplasm bound to a number of accessory proteins such as heat shock protein (hsp)-90 and peptidyl-prolyl isomerases known as FK-binding proteins. However, following binding of the GR receptor to glucocorticoids such as dexamethasone, these accessory proteins are released and the ligand-receptor complex translocates into the nucleus. Once there, the GR forms into a homodimer, binds to glucocorticoid responsive elements (GRE) within genomic DNA, leading to an increase (*trans*-activation) in the expression of anti-inflammatory genes or, less commonly, repression of inflammatory genes (*cis*-repression). At present, *cis*-repression is thought to be unlikely to be responsible for the widespread anti-inflammatory response since it has been observed only at high glucocorticoid concentrations. Instead, the actions of glucocorticoids are believed to be primarily mediated through the inhibition of the pro-inflammatory transcription factors, NF-κB and AP-1. This process, entitled *trans*-repression, occurs following both protein-protein interactions between the GR and the individual transcription factors, as well as modulation of chromatin structure [36,37].

Little is known of the role of miRNAs in the innate immune response although recent reports have shown that this is associated with increased miR-146 and miR-155 expression in monocytic cell lines and murine macrophages [38,39]. Interestingly, since two of the predicted targets for miR-146, TRAF6 and IRAK1 are known to be involved in LPS signalling, these authors speculate that miR-146 might regulate inflammation via a classical negative feedback pathway [39]. However, cell based studies often differ significantly from the *in vivo *milieu, which involves the interaction of multiple cell types. For this reason, we have examined the profile of miRNA expression during the innate immune response in mouse lung following aerosilised LPS challenge. In agreement with the report of Taganov *et al *[39], we observed a rapid increase in the profile of miRNA expression and showed that this correlated with the resolution of inflammation. Furthermore, we demonstrate that the actions of glucocorticoids, which are known to potently inhibit the LPS-induced inflammatory response [40,41], are mediated at the transcriptional level and not through changes in miRNA expression.

## Results and discussion

### LPS induces an acute inflammatory response in mouse lung

**Figure 1:**
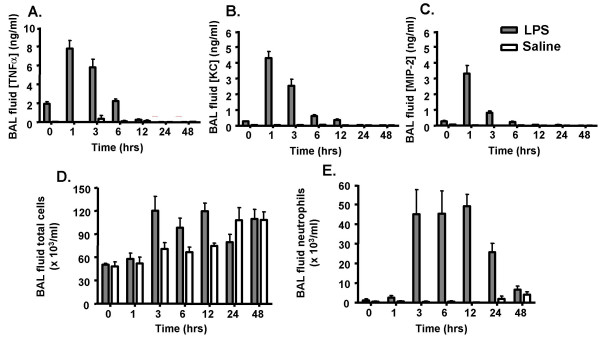
Time course of LPS-induced changes in BAL cytokines and cell infiltrates. Mice were exposed to aerosolised LPS and at the indicated time, the BAL levels of TNF-α (A), KC (B) MIP-2 (C), total leukocyte (D) and neutrophils (E) were determined. The values give are the mean ± SEM obtained from 6 mice.

To investigate the role of miRNAs in the mechanisms of the innate immune response *in vivo*, we employed a LPS-induced model of murine lung inflammation [42]. Exposure of BALB/c mice to aerosolised LPS led to an immediate and dramatic increase in the airway concentration of the pro-inflammatory cytokines and chemokines, TNF-α, KC (the mouse equivalent of IL-8) and MIP-2, as determined in bronchoalveolar lavage (BAL) fluid (Figure 1A–C). In all cases, this response peaked at approximately 1 hr before dropping to essentially baseline levels at 6 – 12 hrs. Examination of the recruitment of inflammatory cells into the airways showed that this lagged behind the release of inflammatory mediators. Thus, an increase in total cell numbers in the bronchoalveolar lavage (BAL) was first seen at 3 hrs (Figure 1D) whilst differential cell counts showed that this increase was mainly attributable to neutrophil recruitment (Figure 1E).

### miRNA expression profile following LPS challenge

From the profile of cytokine release, it appeared that we might determine a role for miRNAs in the LPS-induced innate immune response by measuring mature miRNA levels in the lung tissue over 6 hrs. To this end, the time-dependent changes in the miRNA expression profile in RNA purified from whole mouse lung at 1 hr, 3 hrs and 6 hrs were measured using a novel RT-PCR based approach [43]. To account for RNA loading, all samples were normalised to 18S whilst changes in expression levels were expressed as a fold-difference to time-matched controls in which animals were exposed to aerosolised saline. Among the 156 assays available no appreciable target detection (Ct >38) occurred with the negative controls (cel-miR2, cel-lin-4, ath-miR-159a) or with 13 miRNAs not predicted to exist in mouse according to the Sanger Institute miRNA registry version 7.1 [20,21]. Of the remainder 140 assays, 36 were found to express at a low levels (Ct >30), a feature associated with intermittent loss of exponential PCR product increase which we deemed inappropriate for reliable relative quantification. The order of expression of the remaining 104 miRNAs according to ΔCt is shown in Table S1.

**Figure 2:**
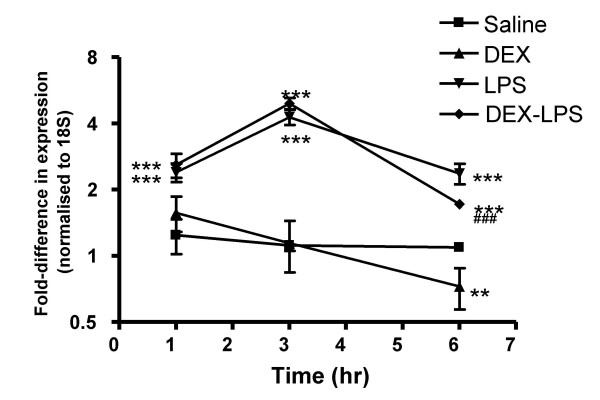
Time-dependent changes in the overall miRNA expression levels. Mice pre-treated intraperitoneally for 1 hr with either saline or dexamethasone, were challenged with either aerosilised saline or LPS. At the indicated time, the mean ± SEM (n = 5) of the change in lung tissue levels for 104 mature miRNAs was determined relative to time-matched saline controls (equivalent to 1). Statistical significance was determined by ANOVA followed by Tukey's post-test where ** = p < 0.01 and *** = p < 0.001 versus saline and ### = p < 0.001 for dexamethasone-LPS versus LPS-only treated animals.

**Figure 3:**
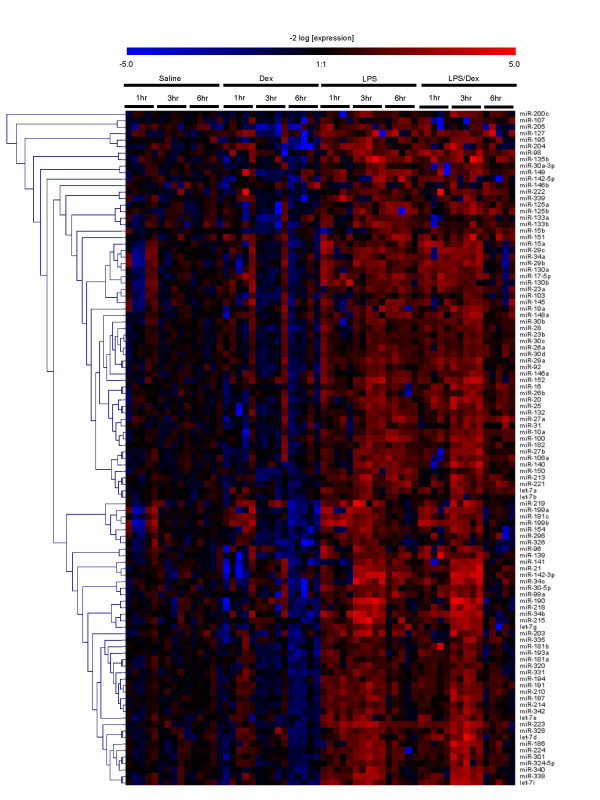
Hierarchically clustered (average linkage) heat map of the time course of LPS- and/or dexamethasone-induced changes in miRNA expression in mouse lung tissue. Mice were exposed to either aerosilised LPS, intraperitoneally administered dexamethasone or were pre-treated with dexamethasone (1 hr) prior to LPS challenge. At the indicated time points following LPS challenge, the levels of 104 mature miRNAs were determined using a TaqMan assay panel. The log_2 _transformants of the fold-change in expression compared to time-matched saline controls are represented.

Exposure to aerosolised LPS resulted in a general elevation in mature miRNA levels at all time points. Examination of the mean increase among all 104 miRNAs (Figure 2), an hierarchically clustered (average linkage) heat map of the miRNA expression profile (Figure 3), as well as the individual miRNAs that were significantly up-regulated (Table S2), showed that this response was time-dependent and peaked at 3 hrs. Thus, the average fold increase above the time-matched saline controls was 2.39 ± 0.23, 4.27 ± 0.33 and 2.37 ± 0.25 at 1 hr, 3 hrs and 6 hrs, respectively (Figure 2). Since little is known of the functional role of miRNAs and given the limitations of the programmes that predict miRNA targets, unlike mRNA expression data, cluster analysis is unlikely to provide reliable information on the potential mechanism of action of miRNA in LPS induced inflammatory response. However, examination of those miRNAs that were significantly up-regulated (p < 0.01) identified 11 miRNAs that were increased at 1 hr, with a peak of 46 miRNAs at 3 hrs which then dropped to 21 miRNAs at 6 hrs (see additional Table S2). Interestingly, in contrast to previous studies that have tended to take a 'snapshot' of miRNA expression by investigating a single time point, these transient changes in the miRNA levels suggest that there is rapid turnover of miRNAs *in vivo*. Although we are presently examining their functional role, a comparison of the time course in miRNA expression and cytokine production leads us to speculate that individual miRNAs might be involved in the resolution rather than the induction of inflammation: the peak in the lung tissue mature miRNA levels at 3 hrs coincides with the drop in production of BAL and tissue TNF-α, KC and MIP-2 (Figure 1). It could be postulated that these time-dependent increases are caused by vasodilation, increased blood flow and neutrophil migration, however this seems unlikely given that the miRNA increases are seen at 1 hr despite the absence of leukocyte infiltration and miRNAs are decreased at 6 hrs even though neutrophil levels are still elevated (Figure 1D/E).

In contrast to previous cell based studies [39] that showed up-regulation of only 3 miRNAs (miR-132, miR-146 and miR-155) following LPS stimulation of the monocytic THP-1 cell line, we observed LPS-induced expression of 46 miRNAs at 3 h. Although these differences could be related to the sensitivity of the methodology used to measure miRNA levels, we believe that this is most likely ascribable to the differences between the *in vitro *and *in vivo *milieus. Thus, the lung contains multiple cell types and exposure to LPS is known to activate a range of intracellular and extracellular pathways.

**Figure 4:**
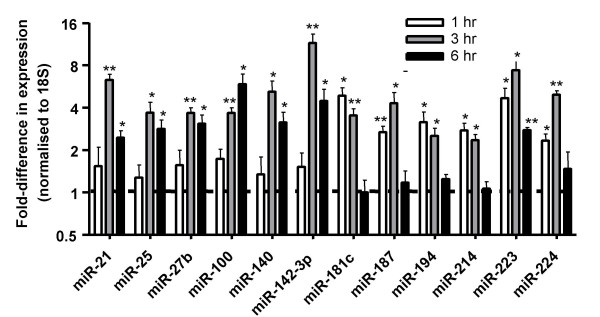
Time dependent, LPS-induced increases in mature miRNA expression levels. At the indicated times, the levels of individual mature miRNAs were determined by TaqMan and were expressed as the fold change ± SEM (n = 5) compared to time-matched saline controls (where saline controls are equivalent to 1). Statistical significance was determined by non-parametric ANOVA followed by Dunn's post-test where * p < 0.01 and ** p < 0.001.

In an attempt to identify individual miRNAs that might be involved in the innate immune response, we found 12 miRNAs whose expression was increased in a time-dependent fashion. From Figure 4, it can be seen that miR-21, -25, -27b, -100, 140, -142-3p, -181c, 187, -194, -214 and -224 were significantly up-regulated at two time points whilst the level of miR-223 was significantly increased at 1, 3 and 6 h. Unlike the LPS-induced response in THP-1 cells and mouse macrophages, we failed to observe up-regulation of miRNA-146 or -155 [38,39]. The reasons behind these differences are presently unknown although they could be a result of multiple factors including the presence of multiple cell types within the lung or be related to the dynamics of the two models. Thus, compared to cell culture models, lungs are likely to be exposed to lower LPS doses over shorter time periods. Future studies might therefore measure miRNA-146 and miRNA-155 expression during chronic inflammatory responses and/or at doses that result in lung injury.

**Figure 5:**
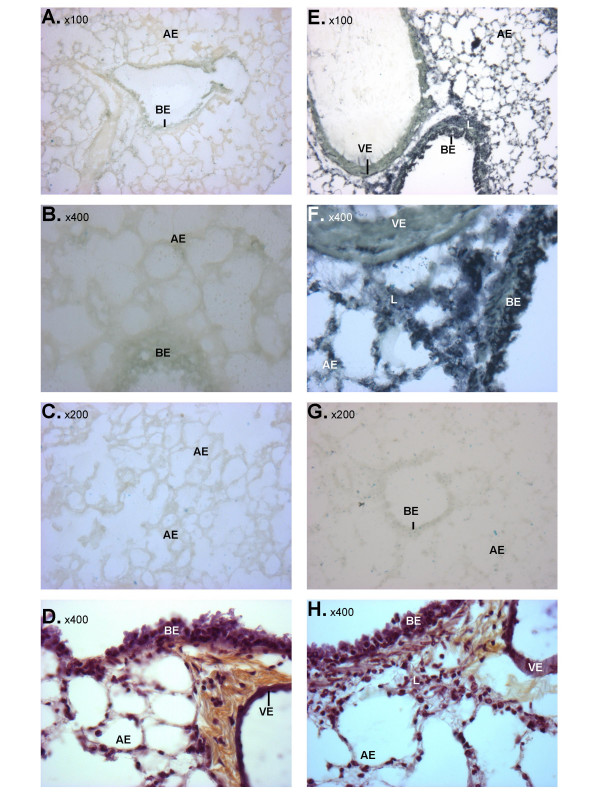
LPS-induced changes in miRNA-223 expression in the mouse lung. Lungs were removed from saline-challenged (A-D) and LPS-challenged (E-H) mice at 3 hrs post-challenge and tissue slices were examined by *in situ *hybridisation using either a miRNA-223-specific (A, B, E, F) or scrambled (C, G) LNA probe, or histologically following cresyl violet staining (D, H). Bronchial (BE) and alveolar (AE) epithelia, as well as the vascular endothelium (VE) and leukocytes (L) are indicated.

To confirm the results obtained with RT-PCR and determine the distribution of miRNA expression in the inflamed mouse lung, we examined the expression of miRNA-223 using *in situ *hybridisation since this was the only miRNA that was significantly up-regulated at all time points. From Figure 5, it can be seen that we observed a prominent increase in miRNA-223 expression in alveolar and bronchial epithelial cells at 3 h following exposure to aerosilised LPS. Furthermore, we observed the migration of inflammatory cells into the bronchioles, most probably neutrophils, which are also stained for miRNA-223. Interestingly, the LPS-induced increases appear to be cell type-specific since we observed very limited changes in miRNA-233 expression within vascular endothelial cells.

### Effect of dexamethasone upon the profile of miRNA expression

**Figure 6:**
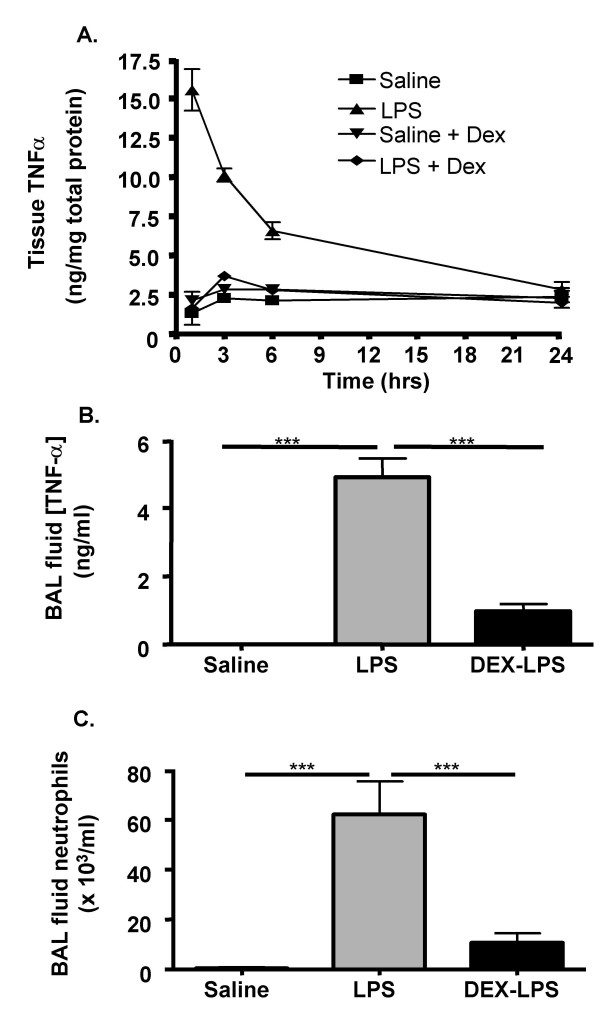
Dexamethasone-mediated inhibition of LPS-induced changes in lung tissue TNF-α, BAL fluid TNF-α and BAL neutrophils. Mice were pre-treated either intraperitoneally (-1 hr – A) or orally (-0.5 hr – B/C) with dexamethasone, then exposed to aerosilised LPS and at the indicated time points the tissue levels of TNF-α (A) or BAL TNF-α (B) were determined by sandwich ELISA and inflammatory neutrophilia (C) was determined by differential cell counting. The values give are the means ± SEM obtained from 6 mice.

To investigate further the role of miRNAs in the innate immune response, it was decided to examine the effect of treatment with dexamethasone, both alone and prior to LPS challenge. As expected, administration of dexamethasone alone had no effect upon basal TNF-α production in lung tissue (Figure 6A). However, pre-treatment with dexamethasome was found to inhibit LPS-induced increases in the tissue and BAL TNF-α levels, as well as BAL neutrophils (Figure 6A–C). In contrast to LPS, examination of the mature miRNA mean expression level (Figure 2) and expression profile (Figure 3) following intraperitoneal administration of dexamethasone alone showed no effect at 1 hrs and 3 hrs post-saline aerosol challenge, but small down-regulation of expression at 6 hrs. Investigation of the extent of miRNA expression downregulation across multiple time points failed to identify individual miRNAs whose basal expression might be sensitive to GR-mediated repression. However, the expression of 1 miRNA (miR-139) and 10 miRNAs (miR-20, -34b, -154, -181c, -187, -204, -296, -301, -340, and -342) were significantly reduced at 3 h and 6 h, respectively.

Given the inhibition of the LPS-induced inflammatory response by glucocorticoids, we ventured to examine whether this might also be mediated through changes in miRNA expression. From figures 2 and 3, it can be seen that pre-treatment with dexamethasone had little effect upon either the mean LPS-induced increases in miRNA expression or the miRNA profile at 1 h and 3 h after aerosolised LPS challenge. In contrast, as with dexamethasone alone, there was a small reduction in overall miRNA expression at 6 h (mean fold changes reduced from 2.36 ± 0.25 to 1.72 ± 0.07 (p < 0.001)). However, the fact that this response was not observed until this extended time point implies that these changes might be secondary to the action of glucocorticoids upon the transcription of protein-encoding genes. Once again we identified a number of miRNAs whose expression appeared to be repressed at a single time point including miR-187 (1 hrs), miR-27b (6 h), miR-29c (6 h), miR-100 (6 h), miR-149 (6 h), miR-150 (6 h) and miR-154 (6 h) (Table S2). Only the expression of miR-154 and miR-187 were found to be inhibited by dexamethasone in both the absence and presence of LPS stimulation. This lack of effect of dexamethasone suggests that LPS-induced increases in miRNA expression are not mediated through activation of pro-inflammatory transcription factors, such as NF-κB and AP-1, which are known to be inhibited by glucocorticoids. Furthermore, since neutrophil migration was attenuated in the presence of dexamethasone, this observation provides further evidence that inflammatory vascular changes and cell migration do not contribute to the LPS-induced changes in miRNA expression (Figure 4B).

## Conclusion

In summary, we have used a novel RT-PCR based approach to measure the differential mature miRNA expression profile during the innate immune response to aerosilised LPS in the mouse lung. Unlike previous studies, which have examined the role of miRNAs during medium and long term responses such as apoptosis/proliferation [23,24], development [25-27], insulin secretion [28] and cholesterol metabolism [29,30], our studies have shown that LPS induced rapid and transient changes in lung miRNA expression and imply that endogenous RNAi might regulate acute phenotypic responses. Although these studies have not identified the functional role of the individual miRNAs over-expressed during LPS-induced lung inflammation, we speculate that a proportion might be involved in the resolution of inflammatory cytokine production since this increased miRNA expression is concomitant to the reduction in the levels of the pro-inflammatory mediators TNF-α, KC and MIP-2. Since the resolution of inflammation is crucial to preventing tissue damage, a range of mechanisms have evolved to regulate this process including negative feedback inhibition of intracellular pathways [42] and the release of anti-inflammatory mediators [44]. Significantly, this report suggests that increased miRNA expression might provide an additional post-transcriptional mechanism for the regulation of inflammation. Indeed, miRNA target prediction databases employing algorithms based upon complementary binding between the miRNA seed region and the miRNA recognition elements within target mRNA 3' UTR identify multiple targets for the miRNAs potentially involved in inflammatory responses. However, at present it is difficult to identify specific targets or a specific mechanism since a number of recent publications have questioned the utility of these algorithms and demonstrated that the biological actions of miRNAs are also influenced by additional factors such as the number of MREs [17], the spacing between MREs [19] and mRNA secondary structure [19]. Nonetheless, bioinformatic investigation of the 3' UTRs of TNF-α, KC and MIP-2 predicts that only TNF-α might contain MRE sites for miR-25 and miR-100 and implies that miRNAs are unlikely to regulate the LPS-induced response through direct action upon the mRNAs of these inflammatory mediators. Instead, as shown by the group of Baltimore *et al *[39], it is more likely that any anti-inflammatory actions of miRNAs are mediated through negative feedback regulation of inflammatory signalling pathways. Finally, to investigate the mechanism that promotes LPS-induced miRNA over-expression we examined the effect of the glucocorticoid dexamethasone. Our data indicates that the anti-inflammatory actions of the glucocorticoid appear to be primarily mediated at the transcriptional level since treatment with dexamethasone alone or prior to LPS exposure had little effect upon the profile of miRNA expression. Interestingly, the fact that dexamethasone had little effect upon the LPS-induced increases in miRNA levels suggests that increased transcription of miRNA genes is not mediated by pro-inflammatory transcription factors such as NF-κB and AP-1.

## Methods

### Animal studies

Male BALB/c mice were obtained from Harlan UK (Bicester, UK) and were allowed to acclimatise for a week prior to use. Food and drink were provided *ad libitum*. All procedures carried out were performed in accordance with the UK 1986 Animals (Scientific Procedures) Act. For studies involving measurement of BAL cytokines and cellular infiltrate, mice were orally dosed with 200 μl volumes of vehicle (1% m/v carboxymethylcellulose, Sigma-Aldrich, Poole, UK) or 1 mg/kg dexamethasone (Sigma-Aldrich) in vehicle 0.5 hrs prior to LPS exposure. For studies of miRNA expression and lung tissue TNF-α, mice were injected intra-peritoneally with 300 μl of either saline or 5 mg/kg dexamethasone in saline, 1 hr prior to aerosol exposure. The mice were challenged with either aerosolised saline or LPS (0.1 mg/ml, *Escherichia coli *serotype 0111:B4, Sigma) for 0.5 hrs using an Ultra-Neb 99 nebuliser (Sunrise Medical Ltd., Wollaston, UK). Animals were sacrificed at set time-points post-challenge with an intra-peritoneal injection of sodium pentobarbitone (200 mg/kg).

### Bronchoalveolar lavage

Immediately following termination, the lungs were exposed and the trachea was cannulated. Lungs were then lavaged three times for 30 sec with 0.3 ml aliquots of room-temperature sterile RPMI media (Invitrogen, Paisley, UK) and the aliquots were pooled. Total and differential cell counts were performed and the levels of TNF-α, KC and MIP-2 in the supernatant were measured using mouse-specific, sandwich, enzyme-linked-immunosorbent assay (ELISA) kits (R&D Systems UK, Oxfordshire, UK), according to the manufacturer's instructions. Optical densities were read on a microplate reader at 450 nm (Spectromax PLUS, Molecular Devices, Sunnyvale, CA, U.S.A.).

### miRNA extraction and measurement

Following exposure to aerosilised LPS in the absence or presence of dexamethasone, the left lobe was removed into 1 ml of RNA *later *(Ambion Europe, Huntington, UK), stored overnight at 4°C and then transferred to -20°C. The right lobe was snap frozen in liquid nitrogen and stored at -70°C prior to measurement of tissue TNF-α levels. Total RNA extractions on the left lobe were carried out using the *mir*Vana™ miRNA isolation kit (Ambion Europe) and an Ultraturrax T18 homogenizer according to the manufacturer's instructions. RNA was eluted in 50 μl RNase-free water (Promega UK, Southampton, UK) and stored at -70°C. RNA content and purity was measured in samples diluted 1/50 with RNase-free water using a BioTek PowerWave XS (SSi Robotics, Tustin, CA, U.S.A.) spectrophotometer (yield (± SD): 1.2 ± 0.4 mg/ml; purity (A_260_/A_280_): 1.9 ± 0.1). miRNA expression profiling was carried out on lung tissue total RNA extracts by two-step Taqman^® ^RT-PCR, normalised to 18S. Reverse transcription was carried out on 5 ng of total RNA in 7.5 μl reactions using the TaqMan^® ^MicroRNA Reverse Transcription Kit (Applied Biosystems, Warrington, UK) according to the manufacturer's instructions [43]. In separate reactions, random hexamers (Applied Biosystems) at a concentration of 10 μM were employed for 18S reverse transcription, whereas miRNA-specific stem-loop primers available in the Applied Biosystems Taqman^® ^MicroRNA Assays, Human Panel Early Access kit were used for target-specific miRNA reverse transcription according to the manufacturer's instructions. Using an Applied Biosystems 7500 Real Time PCR System, 10 μl real time PCR reactions were carried out on 0.7 μl of cDNA using TaqMan^® ^Universal PCR Master Mix, No AmpErase^® ^UNG (Applied Biosystems), Eukaryotic 18S rRNA Endogenous Control primer/probe sets (Applied Biosystems), the miRNA-specific primer/probe sets available in the miRNA Panel kit, and RNase-free water (Promega) according to the manufactuer's instructions. The separate well, 2^-ΔΔ(Ct) ^method [45] was used to determine relative-quantitative levels of individual miRNAs, and these were expressed as the fold-difference to the time-matched saline treatment/challenge group (calibrator).

### Measurement of tissue TNF-α levels

Individual right lung lobes were homogenised in ice-cold Dublecco's phosphate buffered saline (DPBS) (Sigma-Aldrich) at a ratio of 1 ml per 100 mg tissue, using an Ultraturrax T18 homogenizer. The homogenates were then spun in a refrigerated (4°C) tabletop microcentrifuge at 21,000 × g for 20 mins. The supernatants were removed and stored at -20°C. Total protein content was determined by Bradford Assay (Bio-Rad, Hertfordshire, UK) according to the manufacturer's instructions. Tissue TNF-α was measured by sandwich ELISA and normalised for total protein content.

### Histology and *in situ *hybridisation

10 μm thick sections were cut and fixed in 4% paraformaldehyde (Sigma) in PBS for 10 min and then washed three times in DPBS (2 min per wash). Sections were incubated with 10 μg/ml proteinase K (Sigma) for 10 min at room temperature and immediately fixed again in 4% paraformaldehyde. After washing three times in DPBS, sections were pre-hybridised in hybridisation buffer (50% formamide (Sigma), 5× SSC buffer (Sigma), 250 μg/ml yeast RNA (Ambion), 1× Denhardt's solution (Sigma) in DEPC (Sigma) treated water) for 1 hr at room temperature in a humidifying chamber. Locked nucleic acid (LNA) probes (miR223 or miR-scrambled) were pre-incubated at 65°C for 5 min and immediately placed on ice. The buffer was removed and replaced with 2 μM miRNA-specific LNA probe or scrambled probe (Exiqon, Denmark) labelled with digoxygenin (DIG) in hybridisation buffer and incubated in a humidifying chamber at 50°C for 18 hrs. Sections were washed twice for 45 min in stringency buffer (50% formamide, 5× SSC buffer in DEPC-treated water, warmed to 50°C) and three times in DPBS at room temperature. Sections were incubated in blocking buffer (10% sheep serum (Sigma) in PBS) for 1 hr at room temperature and then with sheep anti-DIG fAb fragments (Roche Diagnostics) labelled with alkaline phosphotase (AP) diluted in 10% sheep serum for 2 hr at room temperature. Sections were washed three times in DPBS (2 min each) and then incubated with AP-substrate BCIP/NBT substrate kit (Vector Laboratories, Peterborough, UK) for 12 hrs at room temperature in a humidifying chamber. Sections were washed twice in DPBS and once in water and then mounted with aqueous mounting media (DAKO, Denmark). Histology sections were cut at 8 μm thickness and fixed in 95% ethanol (Sigma) for 10 minutes. Following 2 minute incubations in 75% and 50% ethanol, sections were stained for 1 minute with 100 μl of cresyl violet (Ambion), washed three times in water and mounted with aqueous mounting medium. Representative images were digitally photographed at the indicated magnification.

### Statistics

Statistical changes in cytokine expression were determined with the two-tailed student T-test with α set to 0.05 using Prism 4 for Windows (version 4.03). miRNA expression data was analysed and displayed using Genesis (version 1.7.0) designed by Alexander Sturn and obtained from the Institute of Genomics and Bioinformatics at the Graz Institute of Technology. Using this software package, hierarchical clustering (average linkage) was carried out and significant differences were determined by analysis of variance (ANOVA) followed by Dunn's post-test with α set to 0.01.

## Abbreviations

miRNA; microRNA NF-κB: nuclear factor-κB; AP-1: activator protein-1; LPS: lipopolysaccharide, TNF-α; tumour necrosis factor-α: KC; keratinocyte-derived chemokine: MIP-2; macrophage inflammatory protein-2

## Competing interests

The author(s) declare that they have no competing interests.

## Authors' contributions

SAM contributed to the experimental design, miRNA expression profiling, analysis and intepretation of data and preparation of the manuscript; MMP measured cytokine levels in lung and BAL, AEW performed the histology and *in situ *hybridisation studies; MAB and MGB contributed to the experimental design; MAL contributed to the concept, design, interpretation and preparation of the manuscript. All authors have read and approved the final manuscript.

## Supplementary Material

Additional file 1 Order of miRNA expression in mouse lung. The relative quantities of miRNAs in saline treated mouse lung (1 hr) was determined by Taqman RT-PCR and expressed as the difference in Ct compared to 18S (ΔCt): the lower the ΔCt value, the higher the expression. Values are the mean ± SEM obtained from 5 mice. Click here for file

Additional file 2 Time dependent changes in lung tissue mature miRNA levels following exposure to LPS with or without dexamethasone pre-treatment. The miRNAs whose expression is significantly changed in the mouse lung in response to LPS challenge and/or dexamethasone pre-treatment, are expressed as a fold-difference of the time-matched saline-treated controls. The effect of treatment/challenge compared to saline administration on individual miRNA expression was analysed statistically with significance set at p < 0.01 (p vs. saline). Statistically significant changes due to dexamethasone pre-treatment in LPS-challenged animals (p vs. LPS column in DEX-LPS treatment group) are also reported. Click here for file
