# Supplementary material for: Expression profiling in vivo demonstrates rapid changes in lung microRNA levels following lipopolysaccharide-induced inflammation but not in the anti-inflammatory action of glucocorticoids
Source: BMC Genomics. 2007 Jul 17;8:240. doi: 10.1186/1471-2164-8-240 (PMC1940008; doi:10.1186/1471-2164-8-240)
Supplement: Additional file 1 — Order of miRNA expression in mouse lung. The relative quantities of miRNAs in saline treated mouse lung (1 hr) was determined by Taqman RT-PCR and expressed as the difference in Ct compared to 18S (ΔCt): the lower the ΔCt value, the higher the expression. Values are the mean ± SEM obtained from 5 mice. [file 1471-2164-8-240-S1.doc]

| **Target** | **Average DCt to 18S** | **stdev** |
| --- | --- | --- |
| miR-146b | 3.39 | 0.51 |
| miR-30b | 3.60 | 1.21 |
| miR-26a | 3.64 | 0.68 |
| miR-30c | 3.99 | 0.81 |
| miR-16 | 4.28 | 0.58 |
| miR-29a | 4.54 | 0.75 |
| let-7b | 5.09 | 0.75 |
| miR-29c | 5.12 | 2.08 |
| miR-21 | 5.54 | 0.75 |
| miR-125b | 5.63 | 0.73 |
| miR-30d | 5.66 | 0.78 |
| miR-125a | 5.70 | 0.72 |
| miR-142-3p | 5.70 | 0.29 |
| miR-27b | 5.99 | 0.76 |
| miR-26b | 6.25 | 0.69 |
| miR-145 | 6.28 | 1.76 |
| miR-23b | 6.29 | 0.71 |
| let-7a | 6.30 | 0.71 |
| miR-30e-5p | 6.37 | 0.84 |
| let-7g | 6.37 | 0.44 |
| miR-23a | 6.39 | 1.62 |
| miR-146a | 6.62 | 0.59 |
| miR-29b | 6.73 | 2.46 |
| miR-130a | 6.77 | 1.45 |
| miR-27a | 6.82 | 1.10 |
| miR-103 | 6.87 | 1.54 |
| miR-195 | 6.88 | 0.29 |
| miR-99a | 6.98 | 0.81 |
| miR-133a | 6.98 | 1.22 |
| miR-15b | 7.09 | 0.98 |
| miR-30a-3p | 7.12 | 0.78 |
| miR-200c | 7.18 | 0.83 |
| miR-133b | 7.27 | 1.20 |
| let-7d | 7.34 | 0.85 |
| miR-181a | 7.35 | 0.99 |
| miR-25 | 7.35 | 0.77 |
| miR-100 | 7.43 | 0.73 |
| miR-150 | 7.50 | 0.86 |
| miR-141 | 7.56 | 0.89 |
| miR-140 | 7.81 | 0.57 |
| miR-223 | 7.84 | 0.44 |
| miR-92 | 8.04 | 0.97 |
| miR-181b | 8.14 | 0.22 |
| let-7i | 8.17 | 1.13 |
| miR-320 | 8.21 | 0.96 |
| miR-19a | 8.26 | 1.61 |
| miR-191 | 8.35 | 0.80 |
| miR-10a | 8.69 | 0.56 |
| miR-34a | 8.79 | 2.45 |
| miR-218 | 8.84 | 0.48 |
| miR-148a | 8.93 | 0.66 |
| miR-20 | 9.05 | 1.08 |
| miR-31 | 9.06 | 0.53 |
| miR-203 | 9.07 | 1.07 |
| miR-222 | 9.15 | 0.48 |
| miR-15a | 9.16 | 1.46 |
| miR-221 | 9.24 | 0.37 |
| miR-214 | 9.29 | 0.48 |
| miR-142-5p | 9.33 | 0.83 |
| miR-152 | 9.49 | 0.98 |
| miR-34c | 9.52 | 0.83 |
| miR-106a | 9.56 | 1.00 |
| miR-186 | 9.63 | 0.58 |
| miR-342 | 9.72 | 0.61 |
| miR-328 | 9.84 | 0.76 |
| miR-17-5p | 9.87 | 1.26 |
| miR-335 | 9.93 | 0.36 |
| miR-301 | 9.97 | 0.73 |
| miR-130b | 10.08 | 2.04 |
| miR-210 | 10.12 | 0.66 |
| miR-199a | 10.45 | 2.98 |
| miR-194 | 10.50 | 0.42 |
| miR-187 | 10.75 | 0.29 |
| miR-182 | 10.90 | 0.12 |
| miR-199b | 11.02 | 3.14 |
| miR-324-5p | 11.07 | 0.43 |
| miR-98 | 11.09 | 0.57 |
| miR-331 | 11.14 | 0.65 |
| miR-205 | 11.17 | 1.77 |
| miR-193a | 11.32 | 0.31 |
| miR-339 | 11.35 | 1.55 |
| miR-28 | 11.50 | 0.74 |
| let-7e | 11.55 | 1.26 |
| miR-149 | 12.29 | 0.95 |
| miR-181c | 12.31 | 1.52 |
| miR-132 | 12.49 | 0.76 |
| miR-326 | 12.64 | 1.50 |
| miR-296 | 12.65 | 1.23 |
| miR-107 | 12.70 | 0.87 |
| miR-338 | 12.82 | 1.16 |
| miR-213 | 13.12 | 0.84 |
| miR-127 | 13.25 | 1.28 |
| miR-204 | 13.43 | 1.20 |
| miR-96 | 13.51 | 1.31 |
| miR-139 | 13.61 | 0.88 |
| miR-34b | 13.84 | 0.41 |
| miR-340 | 14.24 | 0.17 |
| miR-135b | 14.28 | 1.25 |
| miR-215 | 14.34 | 1.03 |
| miR-151 | 14.41 | 0.59 |
| miR-224 | 14.44 | 0.74 |
| miR-190 | 14.54 | 0.75 |
| miR-154 | 15.64 | 2.16 |
| miR-219 | 16.07 | 1.28 |
