# Supplementary material for: Expression profiling in vivo demonstrates rapid changes in lung microRNA levels following lipopolysaccharide-induced inflammation but not in the anti-inflammatory action of glucocorticoids
Source: BMC Genomics. 2007 Jul 17;8:240. doi: 10.1186/1471-2164-8-240 (PMC1940008; doi:10.1186/1471-2164-8-240)
Supplement: Additional file 2 — Time dependent changes in lung tissue mature miRNA levels following exposure to LPS with or without dexamethasone pre-treatment. The miRNAs whose expression is significantly changed in the mouse lung in response to LPS challenge and/or dexamethasone pre-treatment, are expressed as a fold-difference of the time-matched saline-treated controls. The effect of treatment/challenge compared to saline administration on individual miRNA expression was analysed statistically with significance set at p < 0.01 (p vs. saline). Statistically significant changes due to dexamethasone pre-treatment in LPS-challenged animals (p vs. LPS column in DEX-LPS treatment group) are also reported. [file 1471-2164-8-240-S2.doc]

| **Dexamethasone** | | | | | | | | | |  |  |  |
| --- | --- | --- | --- | --- | --- | --- | --- | --- | --- | --- | --- | --- |
|  | **1hr** | | | **3hr** | | | **6hr** | | |  |  |  |
| **Target** | **Mean** | **SEM** | **p vs saline** | **Mean** | **SEM** | **p vs saline** | **Mean** | **SEM** | **p vs saline** |  |  |  |
| miR-20 | 0.79 | 0.29 |  | 1.75 | 0.87 |  | 0.51 | 0.08 | 0.0089 |  |  |  |
| miR-34b | 2.46 | 1.29 |  | 1.80 | 0.88 |  | 0.27 | 0.05 | 0.0033 |  |  |  |
| miR-139 | 1.58 | 1.02 |  | 0.39 | 0.10 | 0.0068 | 0.69 | 0.20 |  |  |  |  |
| miR-154 | 2.16 | 1.07 |  | 0.62 | 0.10 |  | 0.41 | 0.11 | 0.0025 |  |  |  |
| miR-181c | 3.38 | 0.64 |  | 1.06 | 0.16 |  | 0.39 | 0.11 | 0.0039 |  |  |  |
| miR-187 | 1.06 | 0.25 |  | 0.95 | 0.13 |  | 0.49 | 0.08 | 0.0036 |  |  |  |
| miR-296 | 1.37 | 0.29 |  | 0.45 | 0.05 |  | 0.46 | 0.12 | 0.0062 |  |  |  |
| miR-301 | 1.05 | 0.28 |  | 1.06 | 0.12 |  | 0.46 | 0.04 | 0.0051 |  |  |  |
| miR-340 | 0.77 | 0.14 |  | 0.81 | 0.09 |  | 0.48 | 0.10 | 0.0060 |  |  |  |
| miR-342 | 1.53 | 0.25 |  | 0.95 | 0.18 |  | 0.43 | 0.07 | 0.0033 |  |  |  |
|  |  |  |  |  |  |  |  |  |  |  |  |  |
| **LPS** | | | | | | | | | |  |  |  |
|  | **1hr** | | | **3hr** | | | **6hr** | | |  |  |  |
| **Target** | **Mean** | **SEM** | **p vs saline** | **Mean** | **SEM** | **p vs saline** | **Mean** | **SEM** | **p vs saline** |  |  |  |
| miR-10a | 1.77 | 0.49 |  | 2.38 | 0.37 |  | 3.60 | 0.66 | 0.0058 |  |  |  |
| miR-15a | 4.54 | 1.70 |  | 2.23 | 0.24 | 0.0017 | 2.14 | 0.71 |  |  |  |  |
| miR-15b | 0.58 | 0.19 |  | 1.63 | 0.50 |  | 2.81 | 0.48 | 0.0058 |  |  |  |
| miR-20 | 1.69 | 0.65 |  | 4.47 | 0.67 | 0.0012 | 2.18 | 0.37 |  |  |  |  |
| miR-21 | 1.54 | 0.55 |  | 6.28 | 0.62 | 0.0001 | 2.45 | 0.29 | 0.0046 |  |  |  |
| miR-23a | 1.42 | 0.30 |  | 2.56 | 0.33 | 0.0099 | 2.97 | 0.54 |  |  |  |  |
| miR-23b | 1.54 | 0.45 |  | 2.05 | 0.27 |  | 1.94 | 0.10 | 0.0021 |  |  |  |
| miR-25 | 1.27 | 0.30 |  | 3.69 | 0.68 | 0.0070 | 2.81 | 0.46 | 0.0074 |  |  |  |
| miR-26b | 3.06 | 0.54 | 0.0083 | 2.86 | 0.65 |  | 1.10 | 0.32 |  |  |  |  |
| miR-27b | 1.56 | 0.43 |  | 3.66 | 0.34 | 0.0001 | 3.08 | 0.46 | 0.0021 |  |  |  |
| miR-28 | 2.00 | 0.92 |  | 2.30 | 0.30 | 0.0067 | 1.53 | 0.24 |  |  |  |  |
| miR-29a | 2.45 | 0.53 |  | 3.58 | 0.64 | 0.0043 | 2.75 | 0.55 |  |  |  |  |
| miR-29b | 3.11 | 0.79 |  | 4.84 | 0.73 | 0.0014 | 2.19 | 0.28 |  |  |  |  |
| miR-29c | 3.53 | 1.34 |  | 4.42 | 0.48 | 0.0002 | 2.06 | 0.33 |  |  |  |  |
| miR-30b | 1.71 | 0.50 |  | 2.98 | 0.51 | 0.0074 | 1.71 | 0.11 |  |  |  |  |
| miR-30d | 1.88 | 0.34 |  | 2.29 | 0.26 | 0.0033 | 2.59 | 0.52 |  |  |  |  |
| miR-30e-5p | 3.35 | 0.76 |  | 3.44 | 0.31 | 0.0011 | 1.77 | 0.44 |  |  |  |  |
| miR-34a | 1.52 | 0.56 |  | 3.06 | 0.59 |  | 3.50 | 0.41 | 0.0004 |  |  |  |
| miR-34b | 5.05 | 1.68 |  | 10.11 | 1.78 | 0.0011 | 1.79 | 0.29 |  |  |  |  |
| miR-92 | 2.13 | 0.59 |  | 2.44 | 0.32 | 0.0063 | 1.34 | 0.19 |  |  |  |  |
| miR-98 | 2.00 | 0.53 |  | 4.56 | 0.91 | 0.0076 | 3.19 | 0.78 |  |  |  |  |
| miR-99a | 1.68 | 0.29 |  | 3.77 | 0.63 | 0.0041 | 3.57 | 1.25 |  |  |  |  |
| miR-100 | 1.73 | 0.29 |  | 3.66 | 0.34 | 0.0001 | 5.88 | 1.05 | 0.0017 |  |  |  |
| miR-106a | 1.42 | 0.31 |  | 3.38 | 0.47 | 0.0022 | 3.73 | 0.88 |  |  |  |  |
| miR-125a | 1.27 | 0.21 |  | 2.16 | 0.31 |  | 4.33 | 0.76 | 0.0034 |  |  |  |
| miR-130a | 2.99 | 1.27 |  | 2.87 | 0.41 | 0.0047 | 1.83 | 0.27 |  |  |  |  |
| miR-132 | 1.36 | 0.16 |  | 2.53 | 0.56 |  | 2.93 | 0.33 | 0.0007 |  |  |  |
| miR-133a | 1.77 | 0.60 |  | 2.65 | 0.42 |  | 2.73 | 0.31 | 0.0056 |  |  |  |
| miR-133b | 1.97 | 0.59 |  | 4.99 | 1.01 | 0.0089 | 2.99 | 0.41 |  |  |  |  |
| miR-139 | 3.24 | 1.20 |  | 2.95 | 0.15 | 0.0001 | 2.91 | 0.80 |  |  |  |  |
| miR-140 | 1.34 | 0.44 |  | 5.19 | 0.98 | 0.0037 | 3.14 | 0.58 | 0.0074 |  |  |  |
| miR-141 | 0.54 | 0.11 |  | 4.52 | 0.62 | 0.0007 | 2.29 | 0.62 |  |  |  |  |
| miR-142-3p | 1.52 | 0.39 |  | 11.52 | 1.82 | 0.0007 | 4.46 | 0.95 | 0.0077 |  |  |  |
| miR-145 | 2.27 | 0.86 |  | 1.55 | 0.30 |  | 3.64 | 0.59 | 0.0037 |  |  |  |
| miR-146a | 1.78 | 0.61 |  | 2.64 | 0.59 |  | 2.59 | 0.41 | 0.0067 |  |  |  |
| miR-148a | 1.62 | 0.39 |  | 2.53 | 0.54 |  | 3.62 | 0.60 | 0.0028 |  |  |  |
| miR-149 | 3.06 | 0.90 |  | 3.49 | 0.86 |  | 4.78 | 0.60 | 0.0005 |  |  |  |
| miR-150 | 1.83 | 0.34 |  | 2.66 | 0.65 |  | 4.16 | 0.46 | 0.0002 |  |  |  |
| miR-152 | 2.52 | 0.80 |  | 6.30 | 1.62 |  | 3.04 | 0.53 | 0.0079 |  |  |  |
| miR-154 | 1.99 | 0.63 |  | 4.35 | 1.04 |  | 1.80 | 0.22 | 0.0097 |  |  |  |
| miR-181c | 4.87 | 0.67 | 0.0079 | 3.51 | 0.42 | 0.0004 | 1.00 | 0.22 |  |  |  |  |
| miR-182 | 1.47 | 0.11 | 0.0030 | 2.99 | 0.83 |  | 2.17 | 0.41 |  |  |  |  |
| miR-186 | 1.45 | 0.53 |  | 4.86 | 0.84 | 0.0026 | 1.70 | 0.47 |  |  |  |  |
| miR-187 | 2.68 | 0.27 | 0.0004 | 4.30 | 0.83 | 0.0054 | 1.17 | 0.25 |  |  |  |  |
| miR-190 | 1.82 | 0.35 |  | 14.22 | 2.12 | 0.0003 | 1.50 | 0.33 |  |  |  |  |
| miR-191 | 3.03 | 0.37 | 0.0029 | 4.39 | 1.17 |  | 1.86 | 0.51 |  |  |  |  |
| miR-193a | 2.57 | 0.56 |  | 2.74 | 0.28 | 0.0005 | 1.18 | 0.35 |  |  |  |  |
| miR-194 | 3.15 | 0.58 | 0.0070 | 2.52 | 0.33 | 0.0028 | 1.24 | 0.10 |  |  |  |  |
| miR-199a | 5.35 | 2.08 |  | 4.12 | 0.81 | 0.0056 | 1.26 | 0.17 |  |  |  |  |
| miR-199b | 8.94 | 1.64 |  | 3.72 | 0.75 | 0.0080 | 1.32 | 0.28 |  |  |  |  |
| miR-203 | 2.91 | 0.90 |  | 7.60 | 1.62 | 0.0039 | 2.48 | 0.76 |  |  |  |  |
| miR-210 | 3.25 | 0.65 |  | 3.20 | 0.55 | 0.0066 | 1.85 | 0.49 |  |  |  |  |
| miR-214 | 2.75 | 0.34 | 0.0017 | 2.35 | 0.23 | 0.0019 | 1.06 | 0.13 |  |  |  |  |
| miR-218 | 1.34 | 0.25 |  | 6.25 | 0.99 | 0.0012 | 1.07 | 0.28 |  |  |  |  |
| miR-223 | 4.68 | 0.81 | 0.0022 | 7.37 | 1.14 | 0.0012 | 2.76 | 0.13 | 0.0001 |  |  |  |
| miR-224 | 2.33 | 0.26 | 0.0068 | 4.94 | 0.33 | 0.0001 | 1.47 | 0.47 |  |  |  |  |
| miR-301 | 1.77 | 0.27 |  | 5.46 | 0.82 | 0.0009 | 1.95 | 0.46 |  |  |  |  |
| miR-324-5p | 1.92 | 0.42 |  | 3.39 | 0.34 | 0.0002 | 1.52 | 0.29 |  |  |  |  |
| miR-328 | 2.58 | 0.80 |  | 3.43 | 0.38 | 0.0019 | 1.67 | 0.26 |  |  |  |  |
| miR-331 | 1.74 | 0.23 |  | 3.24 | 0.54 | 0.0071 | 1.42 | 0.16 |  |  |  |  |
| miR-338 | 2.71 | 0.16 |  | 7.61 | 0.59 | 0.0001 | 1.08 | 0.22 |  |  |  |  |
| miR-340 | 1.94 | 0.25 | 0.0064 | 5.21 | 1.34 |  | 1.44 | 0.38 |  |  |  |  |
| miR-342 | 2.45 | 0.53 |  | 3.85 | 0.70 | 0.0048 | 1.44 | 0.37 |  |  |  |  |
| let-7g | 2.74 | 0.41 | 0.0044 | 3.76 | 0.88 |  | 2.83 | 1.06 |  |  |  |  |
| let-7i | 3.91 | 0.93 |  | 4.77 | 0.92 | 0.0064 | 1.53 | 0.34 |  |  |  |  |
|  |  |  |  |  |  |  |  |  |  |  |  |  |
| **Dexamethasone-LPS** | | | | | | | | | | | | |
|  | **1hr** | | | | **3hr** | | | | **6hr** | | | |
| **Target** | **Mean** | **SEM** | **p vs saline** | **p vs LPS** | **Mean** | **SEM** | **p vs saline** | **p vs LPS** | **Mean** | **SEM** | **p vs saline** | **p vs LPS** |
| let-7a | 2.18 | 0.63 |  |  | 4.17 | 0.83 | 0.0055 |  | 2.16 | 0.22 | 0.0046 |  |
| miR-10a | 2.38 | 0.34 | 0.0078 |  | 4.07 | 0.60 | 0.0012 |  | 2.34 | 0.13 | 0.0004 |  |
| miR-15a | 6.12 | 1.40 |  |  | 4.69 | 0.89 | 0.0035 |  | 1.28 | 0.34 |  |  |
| miR-16 | 2.43 | 0.71 |  |  | 5.43 | 0.71 | 0.0003 |  | 2.80 | 0.59 |  |  |
| miR-17-5p | 4.46 | 1.28 |  |  | 3.48 | 0.49 | 0.0039 |  | 2.15 | 0.65 |  |  |
| miR-19a | 1.49 | 0.32 |  |  | 5.79 | 0.77 | 0.0004 |  | 1.88 | 0.47 |  |  |
| miR-20 | 3.29 | 1.10 |  |  | 7.52 | 1.28 | 0.0010 |  | 2.07 | 0.39 |  |  |
| miR-21 | 2.36 | 0.22 | 0.0022 |  | 10.03 | 2.93 |  |  | 1.81 | 0.41 |  |  |
| miR-23a | 3.13 | 0.72 |  |  | 2.66 | 0.32 | 0.0067 |  | 1.97 | 0.56 |  |  |
| miR-26a | 1.94 | 0.47 |  |  | 2.53 | 0.28 | 0.0013 |  | 2.08 | 0.46 |  |  |
| miR-26b | 2.68 | 0.42 | 0.0077 |  | 3.86 | 1.01 |  |  | 1.87 | 0.35 |  |  |
| miR-27a | 1.81 | 0.56 |  |  | 4.48 | 0.74 | 0.0025 |  | 4.50 | 1.20 |  |  |
| miR-27b | 1.59 | 0.44 |  |  | 6.66 | 1.08 | 0.0008 |  | 1.43 | 0.12 |  | 0.0082 |
| miR-28 | 2.48 | 0.52 |  |  | 3.26 | 0.42 | 0.0012 |  | 1.66 | 0.27 |  |  |
| miR-29a | 3.35 | 0.83 |  |  | 4.83 | 0.93 | 0.0036 |  | 1.16 | 0.13 |  |  |
| miR-29b | 4.55 | 0.75 |  |  | 5.35 | 0.93 | 0.0023 |  | 1.05 | 0.26 |  |  |
| miR-29c | 4.73 | 1.31 |  |  | 5.71 | 0.51 | 0.0001 |  | 0.67 | 0.14 |  | 0.0046 |
| miR-30a-3p | 1.41 | 0.64 |  |  | 3.22 | 0.51 | 0.0043 |  | 1.40 | 0.15 |  |  |
| miR-30b | 2.43 | 0.20 |  |  | 3.85 | 0.68 | 0.0040 |  | 1.50 | 0.16 |  |  |
| miR-30c | 2.23 | 0.31 |  |  | 2.84 | 0.47 | 0.0058 |  | 1.54 | 0.15 |  |  |
| miR-30d | 2.40 | 0.35 |  |  | 2.83 | 0.34 | 0.0014 |  | 2.04 | 0.55 |  |  |
| miR-30e-5p | 2.20 | 0.46 |  |  | 4.27 | 0.69 | 0.0039 |  | 1.06 | 0.26 |  |  |
| miR-34a | 2.50 | 0.66 |  |  | 4.86 | 0.81 | 0.0023 |  | 1.53 | 0.47 |  |  |
| miR-34b | 4.36 | 0.51 | 0.0002 |  | 9.72 | 1.15 | 0.0001 |  | 0.88 | 0.16 |  |  |
| miR-34c | 2.88 | 0.83 |  |  | 9.15 | 2.00 | 0.0037 |  | 1.77 | 0.58 |  |  |
| miR-92 | 2.62 | 0.46 |  |  | 3.95 | 0.67 | 0.0032 |  | 1.19 | 0.19 |  |  |
| miR-98 | 5.31 | 1.15 | 0.0066 |  | 5.10 | 1.18 |  |  | 2.86 | 0.52 |  |  |
| miR-99a | 2.49 | 0.72 |  |  | 5.51 | 1.29 | 0.0098 |  | 1.38 | 0.25 |  |  |
| miR-100 | 2.33 | 0.62 |  |  | 6.31 | 1.03 | 0.0009 |  | 1.93 | 0.33 |  | 0.0071 |
| miR-106a | 1.31 | 0.49 |  |  | 3.75 | 0.46 | 0.0008 |  | 2.54 | 0.49 |  |  |
| miR-125a | 1.31 | 0.30 |  |  | 4.89 | 0.93 | 0.0050 |  | 1.99 | 0.44 |  |  |
| miR-127 | 3.33 | 0.72 |  |  | 2.82 | 0.45 | 0.0099 |  | 6.43 | 3.46 |  |  |
| miR-130a | 3.98 | 0.34 | 0.0029 |  | 3.53 | 0.53 | 0.0027 |  | 0.96 | 0.22 |  |  |
| miR-132 | 1.77 | 0.34 |  |  | 2.96 | 0.33 | 0.0008 |  | 1.88 | 0.37 |  |  |
| miR-140 | 1.49 | 0.37 |  |  | 9.08 | 2.26 | 0.0079 |  | 1.62 | 0.20 |  |  |
| miR-141 | 1.37 | 0.46 |  |  | 7.52 | 0.82 | 0.0001 |  | 1.15 | 0.18 |  |  |
| miR-142-3p | 1.32 | 0.25 |  |  | 13.80 | 1.51 | 0.0001 |  | 1.69 | 0.33 |  |  |
| miR-142-5p | 3.14 | 0.39 | 0.0021 |  | 2.47 | 1.01 |  |  | 0.88 | 0.35 |  |  |
| miR-148a | 2.42 | 0.66 |  |  | 5.48 | 1.25 | 0.0081 |  | 2.10 | 0.63 |  |  |
| miR-149 | 2.64 | 1.44 |  |  | 3.73 | 0.90 |  |  | 2.05 | 0.25 |  | 0.0031 |
| miR-150 | 0.99 | 0.10 |  |  | 2.87 | 0.86 |  |  | 1.30 | 0.20 |  | 0.0004 |
| miR-152 | 2.74 | 0.76 |  |  | 8.79 | 2.09 | 0.0062 |  | 1.72 | 0.41 |  |  |
| miR-154 | 1.59 | 0.39 |  |  | 5.36 | 1.10 | 0.0043 |  | 0.72 | 0.21 |  | 0.0071 |
| miR-181b | 5.08 | 3.12 |  |  | 3.00 | 0.38 | 0.0022 |  | 0.90 | 0.21 |  |  |
| miR-182 | 1.45 | 0.12 | 0.0062 |  | 4.67 | 0.65 | 0.0009 |  | 1.71 | 0.40 |  |  |
| miR-186 | 1.72 | 0.29 |  |  | 8.63 | 2.13 | 0.0079 |  | 1.60 | 0.61 |  |  |
| miR-187 | 1.37 | 0.22 |  | 0.0054 | 6.08 | 1.13 | 0.0024 |  | 1.15 | 0.24 |  |  |
| miR-190 | 1.15 | 0.25 |  |  | 16.03 | 3.55 | 0.0034 |  | 0.68 | 0.15 |  |  |
| miR-191 | 2.04 | 0.32 |  |  | 3.94 | 0.53 | 0.0006 |  | 1.92 | 0.25 |  |  |
| miR-194 | 1.92 | 0.17 | 0.0029 |  | 3.34 | 1.06 |  |  | 1.66 | 0.34 |  |  |
| miR-195 | 1.92 | 0.29 |  |  | 4.36 | 0.28 | 0.0001 |  | 1.89 | 0.97 |  |  |
| miR-199a | 4.29 | 1.08 |  |  | 4.58 | 0.55 | 0.0003 |  | 1.12 | 0.29 |  |  |
| miR-199b | 6.76 | 1.25 |  |  | 3.97 | 0.76 | 0.0056 |  | 1.19 | 0.29 |  |  |
| miR-200c | 1.05 | 0.20 |  |  | 4.48 | 0.89 | 0.0048 |  | 1.82 | 0.43 |  |  |
| miR-215 | 2.04 | 0.44 |  |  | 6.58 | 0.77 | 0.0003 |  | 1.04 | 0.35 |  |  |
| miR-218 | 2.07 | 0.71 |  |  | 8.25 | 1.72 | 0.0037 |  | 0.99 | 0.22 |  |  |
| miR-223 | 3.19 | 0.24 | 0.0001 |  | 7.68 | 1.38 | 0.0024 |  | 1.93 | 0.47 |  |  |
| miR-224 | 1.84 | 0.15 |  |  | 5.81 | 1.23 | 0.0064 |  | 1.39 | 0.35 |  |  |
| miR-320 | 1.61 | 0.25 |  |  | 2.56 | 0.30 | 0.0016 |  | 1.10 | 0.24 |  |  |
| miR-328 | 2.10 | 0.57 |  |  | 6.11 | 1.30 | 0.0061 |  | 1.41 | 0.26 |  |  |
| miR-338 | 2.19 | 0.56 |  |  | 7.38 | 0.94 | 0.0002 |  | 0.96 | 0.19 |  |  |
| miR-340 | 1.26 | 0.24 |  |  | 6.11 | 0.79 | 0.0005 |  | 1.61 | 0.46 |  |  |
| let-7e | 2.70 | 0.92 |  |  | 4.38 | 0.69 | 0.0016 |  | 1.40 | 0.20 |  |  |
| let-7g | 1.48 | 0.31 |  |  | 6.67 | 0.94 | 0.0004 |  | 1.28 | 0.38 |  |  |
| let-7i | 2.86 | 0.56 |  |  | 8.88 | 1.05 | 0.0001 |  | 1.74 | 0.75 |  |  |
